# Trends in incidence of pneumothorax in England before, during and after the COVID-19 pandemic (2017–2023): a population-based observational study

**DOI:** 10.1016/j.lanepe.2024.100994

**Published:** 2024-07-01

**Authors:** Xiaomin Zhong, Raph Goldacre, Eva J.A. Morris, Rob J. Hallifax

**Affiliations:** a Applied Health Research Unit, Big Data Institute, Li Ka Shing Centre for Health Information and Discovery, Nuffield Department of Population Health, University of Oxford, Oxford, UK; b Oxford Centre for Respiratory Medicine, Oxford University Hospitals NHS Foundation Trust, Oxford, UK

**Keywords:** Pneumothorax, Incidence, Epidemiology, COVID

## Abstract

**Background:**

COVID-19 is a risk factor for pneumothorax. The pandemic may have influenced healthcare-seeking behaviour for pneumothorax. This study aimed to investigate recent trends in the incidence of pneumothorax in England.

**Methods:**

A population-based epidemiological study was conducted using an English national dataset of hospital admissions (Hospital Episode Statistics) from 2017 to 2023. Record-linkage was used to identify multiple admissions per person and co-morbidity. Pneumothoraces co-occurring with COVID-19 were identified by concurrent COVID-19 diagnostic coding. The pre-pandemic (January 2017–February 2020), pandemic (March-2020–February-2021) and post-pandemic periods (March 2021–March 2023) were compared.

**Findings:**

From 2017 to 2023, there were 72,275 hospital admissions for spontaneous pneumothorax among 59,130 patients. Admissions showed marked variability, peaking in January 2021 when the rate of admissions was about two-thirds higher than that of the pre-pandemic level (Incidence rate ratio [IRR] 1.65, 95% CI: 1.48–1.84). However, when excluding patients with a concurrent COVID-19 diagnosis, the overall trend shifted to a reduction during the pandemic period. Post-pandemic rates were not significantly different from pre-pandemic levels (IRR = 0.96, 95% CI: 0.89–1.04). The incidence of spontaneous pneumothorax was significantly higher in males (rate ratio compared to females: 2.29, 95% CI: 2.19–2.39). However, the trends were consistent in both males and females.

**Interpretation:**

This study highlights a significant peak in COVID-19 related cases but a corresponding trough in non-COVID-related cases (end 2020, early 2021). Despite a previous report of increasing incidence of (non-COVID-related) hospitalised spontaneous pneumothorax over the long-term between 1968 and 2016, we did not observe any continued increase throughout this study period, prompting further investigation into the impact of recent guidelines.

**Funding:**

Authors are supported by the 10.13039/501100000272NIHR Oxford BRC, Li Ka Shing and Robertson Foundations, 10.13039/501100000265MRC, and HDR UK.

**Research in context**

**Evidence before this study**

Before embarking on our study, we conducted comprehensive searches across PubMed for literature published from 1 January 2017 to 31 December 2023. We aimed to identify studies that reported on the incidence and management of spontaneous pneumothorax, both COVID-related and unrelated. Our search terms included combinations of “spontaneous pneumothorax,” “COVID-19 pandemic,” “pneumothorax incidence,” and “pneumothorax management,” without restrictions on language or geographical location. Prior to our analysis, our previous publications reported long-term trends in the incidence and recurrence of inpatient-treated pneumothorax from 1968 to 2016. However, large-scale, up-to-date, comprehensive data encompassing the period of the COVID-19 pandemic were lacking.

**Added value of this study**

Our study represents one of the most extensive analyses to date on the incidence of inpatient-treated spontaneous pneumothorax, including the period affected by the COVID-19 pandemic. By utilising national hospitalisation data from England, we offer a detailed examination of trends before, during, and after the pandemic. Notably, we observed a significant peak in COVID-19 related pneumothoraces during late 2020 and early 2021. Our findings provide novel insights into the impact of the pandemic on the incidence of pneumothorax, distinguishing between primary and secondary spontaneous pneumothorax and highlighting the role of COVID-19 as a contributing factor.

**Implications of all the available evidence**

The evidence presented in our study, combined with existing literature, suggests a significant alteration in the pattern of spontaneous pneumothorax admissions during the COVID-19 pandemic. This shift underscores the necessity for continued vigilance in monitoring pneumothorax cases in the context of COVID-19, considering the potential for increased risk associated with viral infection. Additionally, our findings suggest a plateau in the incidence of non-COVID-related pneumothorax, possibly reflecting changes in clinical management practices and patient behaviour during the pandemic. These insights are crucial for healthcare providers and policymakers in optimising pneumothorax management strategies, particularly in anticipation of potential future public health crises.

## Introduction

Spontaneous pneumothorax is a common pathology. Primary spontaneous pneumothorax conventionally refers to patients with no underlying lung disease, whilst those with established lung pathology are classified as secondary spontaneous pneumothorax. There is a bimodal age-distribution with a first peak aged 15–34 years and an increasing incidence beyond 60 years in both males and females.^1^ Overall incidence of spontaneous pneumothorax is 17–24 per 100,000 population per annum for men and 1–6 per 100,000 for women.^1^, ^2^, ^3^, ^4^, ^5^ In England over the last 50 years, there has been a small gradual increase in the incidence of inpatient admissions for spontaneous pneumothorax.^1^

The COVID-19 pandemic had significant impact on all aspects of healthcare provision and patient behaviour. Retrospective case series data have identified an association between COVID-19 (particularly those patients requiring admission to hospital) and pneumothorax.^6^, ^7^, ^8^ However, up-to-date largescale data on both COVID-associated and non-COVID related spontaneous pneumothorax that include the COVID-19 pandemic period are absent.

The aim of this study was to investigate the recent trends in the incidence of inpatient-treated spontaneous and COVID-related pneumothorax in England in the years covering the COVID-19 pandemic using national hospitalisation data.

## Methods

We conducted a national retrospective observational study of population-based hospitalisation rates for spontaneous pneumothorax in England, spanning January 2017–March 2023.

### Data source

We used an extract of national Hospital Episode Statistics Admitted Patient Care (HES-APC) records, which cover all day case and inpatient care episodes occurring in National Health Service (NHS) hospitals in England. Each discharge record contains a field for the principal reason for the admission (the “primary diagnosis”), along with other secondary/subsidiary diagnoses, coded using the International Classification of Diseases Version 10 (ICD-10).^9^ The extract contained all HES-APC records belonging to individuals admitted to English NHS hospitals between January 2017 and March 2023 with a diagnosis of spontaneous pneumothorax (ICD-10 code J93). If the admission also contained a diagnosis code for iatrogenic pneumothorax (ICD10 J95.8) or indicated traumatic pneumothorax (ICD10 S22, S27 or S42) it was excluded. Elective admissions were also excluded.

To differentiate secondary from primary spontaneous pneumothorax (PSP), for every patient we searched their HES-APC records for any diagnosis of chronic lung disease defined by any of the 73 ICD codes (listed in eTable 1 in the Supplement) as per previous methodology.^1^ The ascertainment period for these comorbidities included the spontaneous pneumothorax record itself and, through record linkage, any known hospital records from the past 10 years or any subsequent records within the following month. Secondary spontaneous pneumothorax (SSP) was defined by the presence of known chronic lung disease, otherwise the pneumothorax was regarded as primary. To keep these definitions consistent throughout the study period, any COVID-19 related spontaneous pneumothorax was excluded from PSP/SSP. COVID-related spontaneous pneumothorax was defined as an admission with a COVID-19 diagnosis (ICD-10 U07.1) recorded alongside spontaneous pneumothorax.

The variables used in these analyses (age, sex, date of admission, method of admission, diagnoses primary and secondary) are all fundamental to the reporting of routine hospital statistics in England, and so missing data for these variables was extremely low: the variable with the most amount of missing data was admission method, which was missing on only 0.05% of all extracted pneumothorax records. This study was conducted on a complete-case dataset.

### Outcomes

The primary outcome was hospital admissions for spontaneous pneumothorax, which were enumerated for each month from January 2017 to March 2023. Transfers between hospitals were counted as a single admission. Monthly admission counts were expressed as rates per 100,000 population using population denominators obtained from the Office for National Statistics.

### Statistical analysis

Monthly admission rates were calculated per 100,000 population by age group, sex, pneumothorax type (overall, PSP, SSP, COVID-related spontaneous pneumothorax). Age was categorised into 5-year age groups for the purposes of age-sex standardisation. Broader age groups (15–34, 35–49, 50–64, ≥65 years) were used for purposes of reporting. We divided the study period into pre-pandemic period (January 2017–February 2020), which was used as the baseline period; the pandemic months (March-2020–February-2021), in which each month was compared individually with the pre-pandemic period; and the post-pandemic period (March 2021–March 2023). A Poisson regression model accounting for over-dispersion was fitted through the monthly rates, adjusting for age (5-year age groups) and sex, with month as a continuous variable to denote the underlying temporal trend, and with each month during the pandemic period and the post pandemic period combined as categorical variables, to obtain incidence rate ratios (IRRs) with 95% confidence intervals, to determine the significance of any temporal change in the hospitalisation rates since March 2020. As we aim to explore temporal trends in overall spontaneous pneumothorax, PSP, and SSP, as well as to discern whether pneumothorax diagnoses were recorded in the principal place of hospitalisation records, we applied the same statistical method across all levels mentioned above. To assess the variations in incidence rate changes before and after the pandemic by sex, we repeated the regression analysis and included an interaction term between gender and the defined period. Additionally, we reported the IRRs stratified by sex. All analyses were performed using R 4.3.0.

### Role of the funding source

The sponsor was not involved in an aspect of the study including: design and conduct of the study; collection, management, analysis, and interpretation of the data; preparation, review, or approval of the manuscript; or decision to submit the manuscript for publication.

## Results

**Fig. 1:**
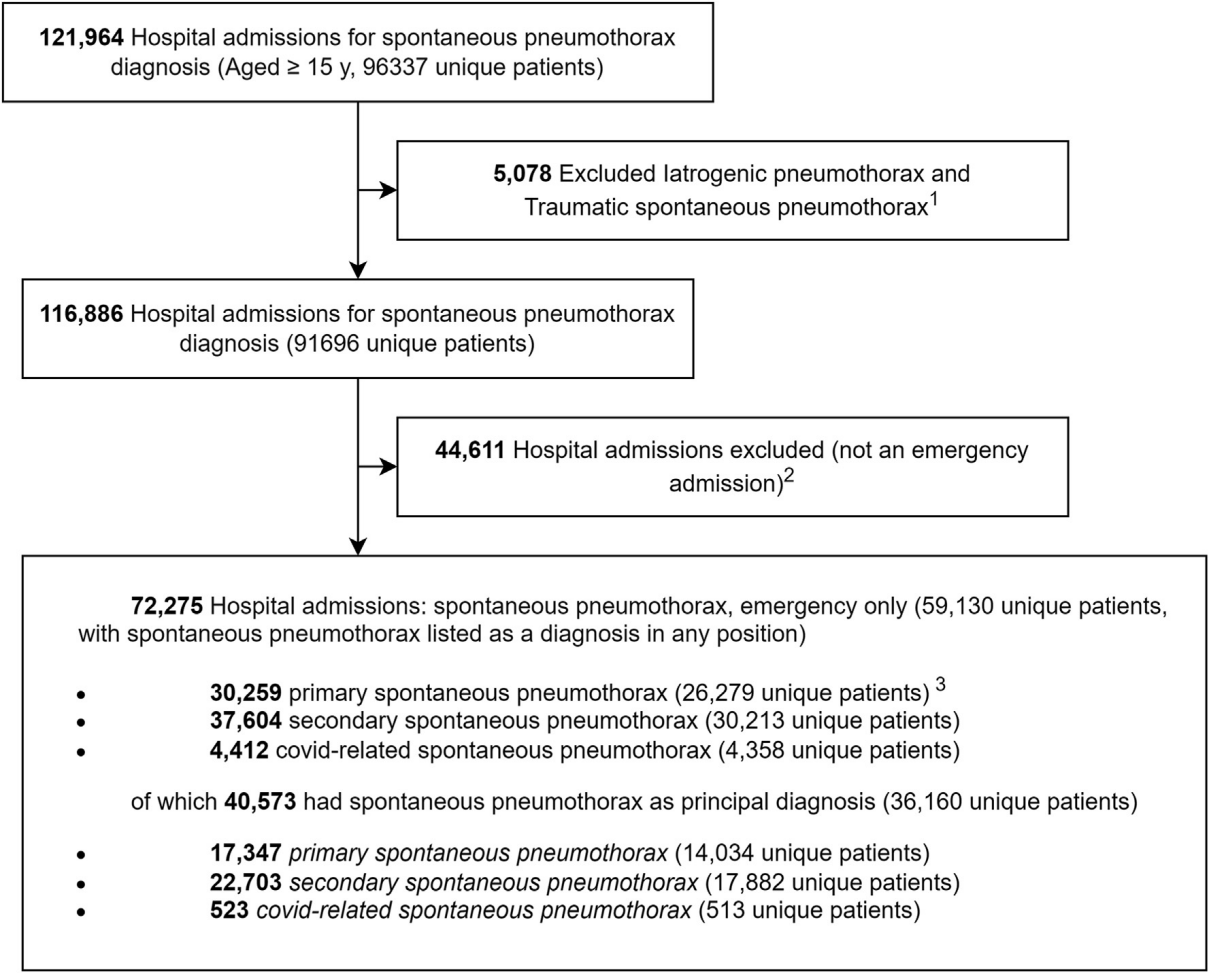
**Flow of pneumothorax admissions from the English Hospital Episode Statistics (HES) from January 2017 through March 2023 for inclusion in the analysis**. ^1^iatrogenic pneumothorax as an important cause to exclude pneumothorax elsewhere on the record as a complication of other procedure, was excluded by removing ICD10 J958. Traumatic spontaneous pneumothoraces were excluded by removing records containing a concurrent diagnosis of fractures of ribs or vertebrae (ICD10 S22), traumatic hemothorax (ICD10 S27) or fracture of upper body (ICD10 S42). ^2^Emergency admissions defined in the HES Data Dictionary—Admitted Patient Care as those for which the admission is “unpredictable and at short notice because of clinical need” and for which admission method was coded 21, 22, 23, 24, 25, 2A, 2B, 2C, 2D or 28. ^3^An admission was regarded as **secondary spontaneous pneumothorax** if the patient had a diagnosis code indicating chronic lung coded either on the same spontaneous pneumothorax record or, using record linkage, on any known previous hospital record in the past 10 years or any record within the next one month. **Primary spontaneous pneumothorax** was defined as any spontaneous pneumothorax admission that was not secondary. To keep the definition consistent, any COVID-19 related spontaneous pneumothorax was excluded from primary/secondary spontaneous pneumothorax. **COVID-related spontaneous pneumothorax** was defined as an admission with a COVID-19 diagnosis (ICD-10 U07.1) recorded alongside spontaneous pneumothorax.

**Fig. 2:**
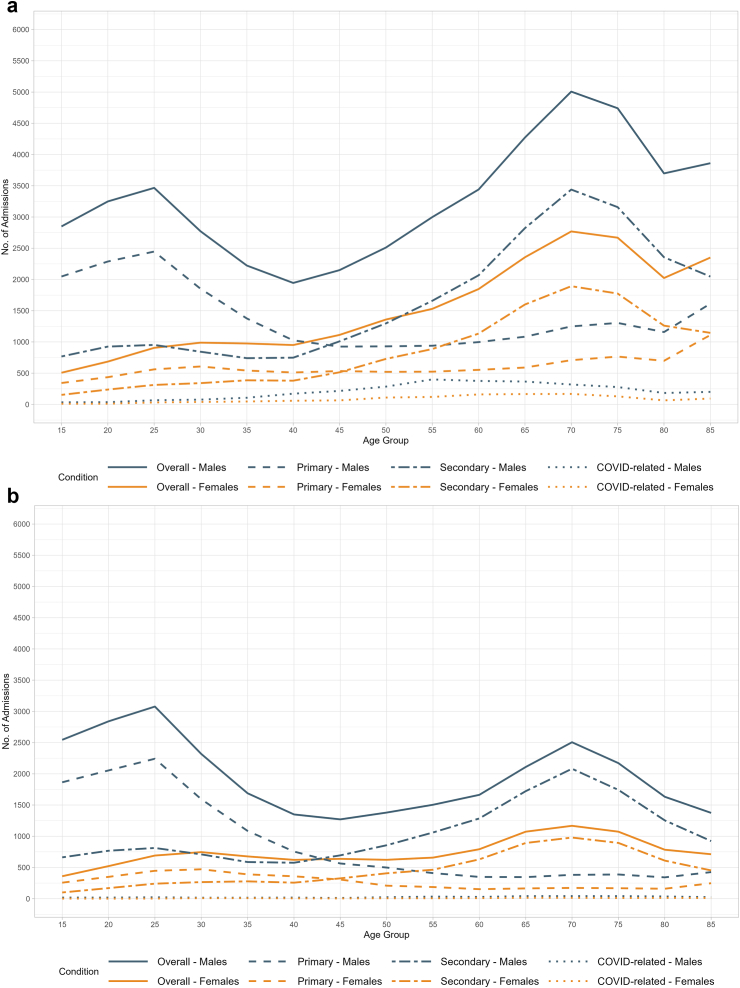
**Primary and secondary spontaneous pneumothorax admissions by age-specific population and sex in England**. a. spontaneous pneumothorax could be any diagnosis (primary/non-primary). b. spontaneous pneumothorax was the principal diagnosis only.

From January 2017 to March 2023, there were 72,275 eligible hospital admissions for spontaneous pneumothorax among 59,130 patients (Fig. 1). The demographic characteristics of these patients are shown in Table 1. 68.1% of these records belonged to men and the mean age at admission was 55.7 years (SD 22.4). Fig. 2 shows the full age-sex distribution of the number of admissions for overall spontaneous pneumothorax, PSP, SSP, and COVID-related spontaneous pneumothorax separately. The breakdown of numbers by pneumothorax types are provided in Table 1, and the equivalent information for where pneumothorax was coded as the principal diagnosis only is provided in eTable 2.

**Table 1:** Spontaneous pneumothorax inpatient admissions in England from January 2017 to March 2023, 15 years and older, by age group and sex.

	Overall spontaneous pneumothorax		Primary spontaneous pneumothorax		Secondary spontaneous pneumothorax		COVID-related Spontaneous Pneumothorax	
	No. of admissions	Mean age (SD)^a^	No. of admissions	Mean age (SD)	No. of admissions	Mean age (SD)	No. of admissions	Mean age (SD)
**Male, overall**	49,182	55.7 (22.4)	21,236	47.7 (23.7)	24,829	61.9 (19.7)	3117	60.8 (16.1)
**Male, by age group, y**	No. of Admissions	%	No. of Admissions	%	No. of Admissions	%	No. of Admissions	%
15–34	11,948	24.5	8354	39.9	3382	13.7	212	6.8
35–49	6318	12.9	3326	15.9	2497	10.1	495	15.9
50–64	8949	18.3	2865	13.7	5023	20.3	1061	34.1
≥65	21,579	44.2	6410	30.6	13,822	55.9	1347	43.2
**Female, overall**	23,028	61.6 (19.8)	9004	57.1 (22.5)	12,747	64.8 (17.4)	1277	62.0 (16.5)
**Female, by age group, y**	No. of Admissions	%	No. of Admissions	%	No. of Admissions	%	No. of Admissions	%
15–34	3008	13.1	1892	21.1	1025	8.1	91	7.1
35–49	3038	13.2	1587	17.7	1280	10.1	171	13.4
50–64	4736	20.6	1598	17.9	2748	21.6	390	30.6
≥65	12,169	53	3872	43.3	7675	60.3	622	48.8

a Mean (SD) was calculated based on the patient age at the admission date of all the hospitalisation records in certain age groups. % represents the percentage of cases among admissions in certain age groups.

### Incidence

#### Spontaneous pneumothorax recorded in any diagnosis position

**Fig. 3:**
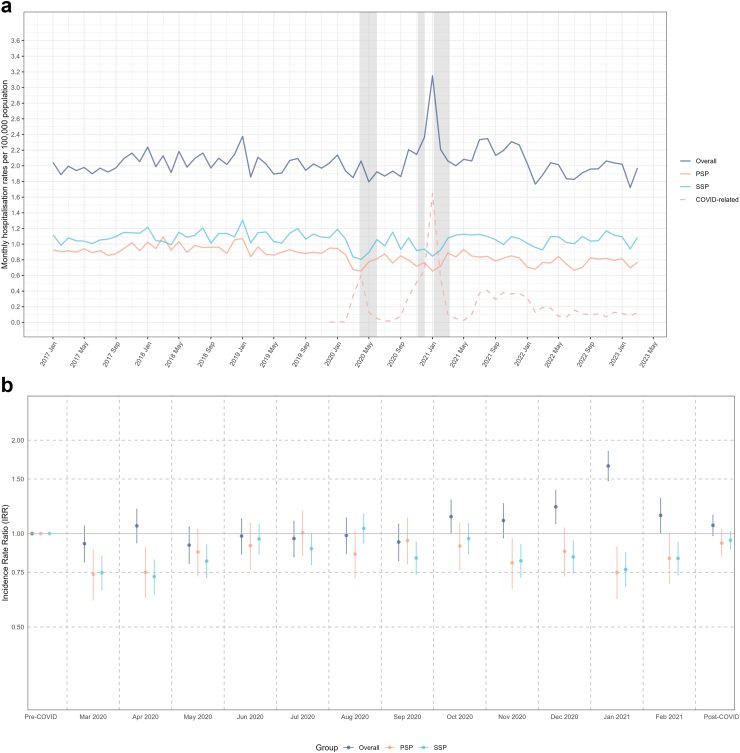
**Hospitalisation rates for spontaneous pneumothorax in England (From January 2017 to March 2023, spontaneous pneumothorax could be any diagnosis (primary/non-primary))**. a. Hospitalisation Rates for Spontaneous Pneumothorax in England. b. Incidence Rate Ratio Comparing Spontaneous Pneumothorax Hospitalization Rates Across Pre-pandemic, Pandemic, and Post-pandemic Periods. ∗The shadow area indicating the periods of national lockdown.

Monthly hospitalisation rates per 100,000 population for spontaneous pneumothorax where the condition was recorded in any diagnosis position are shown in Fig. 3a. The mean monthly hospitalisation rates for the pre-pandemic period were 2.0, 1.1 and 0.9 per 100,000 for spontaneous pneumothorax overall, SSP and PSP, respectively (Fig. 3a). The general trend in inpatient treatment of pneumothorax throughout the pre-pandemic period remained stable, with an overall IRR of 0.999 (95% CI: 0.997–1.000) and a SSP IRR of 0.999 (95% CI: 0.997–1.001). In contrast, the pre-pandemic trend for PSP was significantly downward, as indicated by an IRR of 0.997 (95% CI: 0.995–0.999), although the absolute change was small (from 0.93 per 100,000 population in January 2017 to 0.86 in February 2020). During the national lockdown periods (indicated as shaded areas in Fig. 3a), we observed an increase in admissions for overall spontaneous pneumothorax during the second and third waves of lockdown, with a peak in January 2021: an IRR 1.65 times higher than the pre-pandemic level (Fig. 3b, 95% CI: 1.48–1.84; see eTable 3 for detailed incident rate ratios). This overall increase was driven by COVID-related pneumothorax admissions: in the same months, excluding COVID-related admissions, rates decreased significantly (PSP IRR: 0.75; 95% CI: 0.62–0.91, SSP IRR: 0.77; 95% CI: 0.68–0.87) (Fig. 3b).

#### Spontaneous pneumothorax recorded as principal diagnosis

**Fig. 4:**
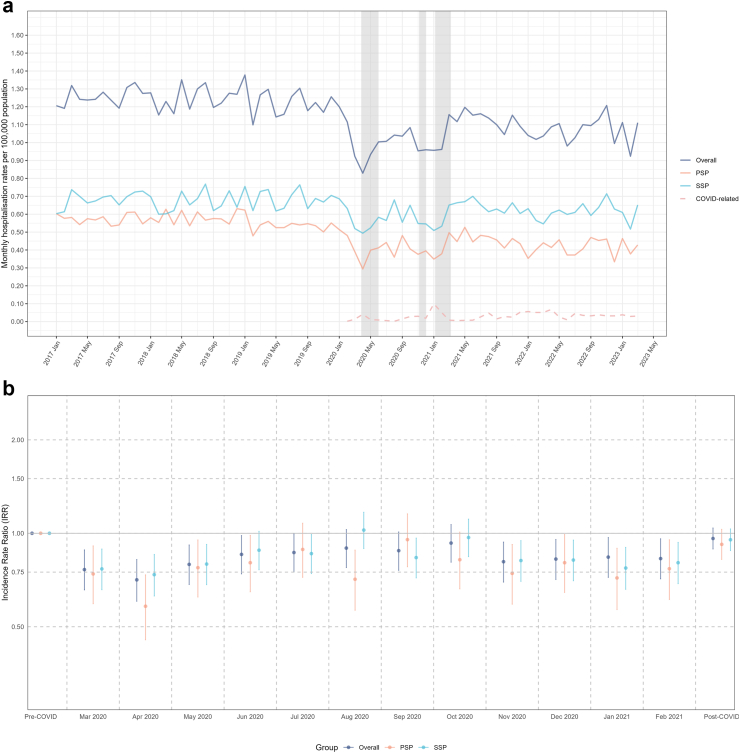
**Hospitalisation rates for spontaneous pneumothorax in England (From January 2017 to March 2023, spontaneous pneumothorax was the principal diagnosis only)**. a. Hospitalisation Rates for Spontaneous Pneumothorax in England. b. Incidence Rate Ratio Comparing Spontaneous Pneumothorax Hospitalization Rates Across Pre-pandemic, Pandemic, and Post-pandemic Periods. ∗The shadow area indicating the periods of national lockdown.

Where spontaneous pneumothorax was recorded only as the principal diagnosis, the mean monthly hospitalisation rates for the pre-pandemic period were 1.2, 0.7 and 0.6 per 100,000 population for spontaneous pneumothorax overall, SSP and PSP, respectively (Fig. 4a). There was a significant decrease in both the first and second lockdowns, with the lowest overall IRR of 0.71 (95% CI: 0.60–0.82) in April 2020 compared to the pre-pandemic level (Fig. 4b). The overall hospitalisation rate of spontaneous pneumothorax in the post-pandemic period was observed to return to pre-pandemic levels (IRR 0.96, 95% CI: 0.89–1.04).

The incidence of spontaneous pneumothorax was significantly higher in males (rate ratio compared to females: 2.29, 95% CI: 2.19–2.39), with consistent results observed among both males and females (eFigure 1–4). There was no significant interaction by sex across different periods.

### Co-morbidities

In the pre-pandemic period the most common principal diagnoses alongside spontaneous pneumothorax were pneumonia (12.8%), pleural effusion (7.4%), chronic obstructive pulmonary disease (5.7%), lung cancer (4.5%) and other diagnoses of sepsis (4.5%) (See Table 2). From the beginning of the pandemic onwards (March 2020), COVID-19 was the most common other diagnosis: 31.2% in the pandemic and 10.9% in the post-pandemic period.

**Table 2:** Top 5 frequent primary diagnoses (alongside with spontaneous pneumothorax) by period: Analysis of admission data from 2017 to 2023.

Rank	Period 1—pre-pandemic	N (%)	Period 2–pandemic	N (%)	Period 3—post-pandemic	N (%)
	type		type		type	
1	Pneumonia, unspecified organism	1878 (12.8)	COVID-19	1942 (31.2)	COVID-19	1224 (10.9)
2	Pleural effusion, not elsewhere classified	1086 (7.4)	Pneumonia, unspecified organism	393 (6.3)	Pneumonia, unspecified organism	1082 (9.6)
3	Other chronic obstructive pulmonary disease	834 (5.7)	Pleural effusion, not elsewhere classified	363 (5.8)	Pleural effusion, not elsewhere classified	718 (6.4)
4	Malignant neoplasm of bronchus and lung	661 (4.5)	Secondary malignant neoplasm of respiratory and digestive organs	220 (3.5)	Other chronic obstructive pulmonary disease	561 (5.0)
5	Other sepsis	655 (4.5)	Malignant neoplasm of bronchus and lung	208 (3.3)	Secondary malignant neoplasm of respiratory and digestive organs	501 (4.5)

The percentage was calculated by dividing the count of the specific ICD-10 code (at the three-character level) by the total count of all diagnoses excluding primary Spontaneous Pneumothorax cases.

Period 1: pre-pandemic (January 2017–February 2020), Period 2: pandemic (March 2020–February 2021), and Period 3: post-pandemic (March 2021–March 2023).

## Discussion

Age-adjusted hospital admission rates in England for spontaneous pneumothorax as principal diagnosis significantly declined during the COVID-19 pandemic across both PSP and SSP. However, in the latter half of the pandemic period, admissions in which spontaneous pneumothorax was coded anywhere on the record increased significantly, driven by admissions in patients whose principal diagnosis was COVID-19. The trends were consistent among males and females, and the incidence in males was more than twice that in females. This result matches the findings in our previous study.^1^

Pre-pandemic, the main other diagnoses were pneumonia and COPD. During the pandemic, unsurprisingly this was overtaken by COVID-19 as the most common other principal diagnosis. However, even in the post-pandemic period COVID-19-related pneumothoraces continued to form a sizeable proportion (10.9%) of overall pneumothorax admissions.

These data represent the largest recent data set of pneumothorax admissions, and the first to specifically analyse admissions pre-, during and post-COVID-19 pandemic. Pneumothorax is a well-reported complication of severe COVID-19 infection and is associated with high mortality rates. Other datasets have focussed on cohorts of patients with COVID-19 to then determine the incidence of pneumothorax in that patient group, rather than the incidence in the whole population. The largest observational study (the ISARIC4C study) included 131,679 patients across 31 hospitals in the UK, reported an overall pneumothorax incidence of 0.97% (1283 out of 131,679), with the highest incidence (8.5%) was found in those patients with COVID-19 who required the highest levels of respiratory support (both non-invasive and invasive ventilation).^8^ Further case series showed a similar incidence of pneumothorax of 0.91% in patients admitted with COVID-19.^6^ and pneumomediastinum (air in the mediastinum) incidence of 0.64%^7^ However, none of these studies reported trends of non-COVID pneumothorax over the time period of the pandemic, or compared to a baseline incidence of non-COVID pneumothorax, limiting understanding of the scale of the challenge the condition poses to the English National Health Service.

Previously, hospital admission rates for spontaneous pneumothorax over the period 1968–2016 had slowly increased.^1^ This was, in part, thought to be attributable to an increase in multiple admissions in the same individuals. However, our updated analyses showed that this increase has not continued, and indeed may be starting to decrease (albeit statistically significant only in PSP, but the overall study period is relatively short).

The pandemic is likely to have had a dual effect on pneumothorax admissions. On one hand, rates may have increased due to complications associated with COVID-19 and the general rise in hospitalisations for the virus itself. On the other hand, admission rates may have been mitigated by changes in management protocols and alterations in patient behaviour, including an aversion to seeking hospital care.^10^^,^^11^ Notably, extended waiting times in ED departments (which has been recently highlighted as a particular challenge in the UK),^12^ coupled with public concerns over potential COVID-19 exposure in hospital settings,^12^ might have discouraged patients from attending hospitals unless absolutely necessary. This reluctance could have further influenced the shift towards ED management of spontaneous pneumothorax, accelerating the adoption of less invasive treatment options and reducing the number of admissions. In addition, pressure from high levels of bed occupancy in the NHS during and after the COVID pandemic may have influenced physician behaviour to prioritise outpatient management. Such dynamics underscore the complex interplay between the pandemic's direct impact on healthcare demand and its indirect influence on healthcare practices and patient preferences.

One of the limitations of the HES-APC dataset is that the data only provide information on patients admitted to hospital. Patients attending the Emergency Department (ED) who are assessed but not admitted are not captured. Therefore, any change in the hospital admission rates could be driven by a change in the proportion being managed as an inpatient versus conservatively or via ambulatory pathways. Published data on the incidence of ED management is limited. One large study using a number of administrative databases from the United States, included data from the Nationwide Emergency Department Sample.^13^ They found that in 2010, 33% of the patients with PSP were treated in ED without admission, but did not report on the management of SSP. The two RCTs of ED management were published in 2020,^14^^,^^15^ so the proportion of PSP patients seen and discharged from ED could potentially have increased from this date onwards, if this data had changed practice immediately. However, a national survey of respiratory physicians in 2021 showed that respondents continued to favour intervention (and hence admission) for patients with large pneumothorax size on chest radiograph.^16^ The impact of the newly published BTS guidelines in terms of a paradigm shift in management is likely to come in the next few years. Therefore the reason for the plateauing of the previously identified increase in incidence as not clear and likely to be multi-factorial.

Moreover, patients with spontaneous pneumothorax usually present to the Emergency Department with pain and/or breathlessness. Previous British Thoracic Society (BTS) guidelines from 2010 suggested that patients with large pneumothorax (as measured on the chest radiograph) should be treated either with a needle aspiration and/or chest drain insertion (for PSP) or directly with chest drain insertion (SSP).^17^ Patients with chest drain require admission to hospital. However, more recently there has been a shift towards outpatient management, for PSP in particular. This change is the result of two large randomised controlled trials (RCTs) of management of PSP. The first RCT in Australia was a trial of entirely conservative management compared to standard care, which showed that 85% of patients in the conservative management arm were managed as outpatients without requiring intervention.^14^ This trial has be criticised because of the high screen-failure rates for recruitment and relatively low patients symptom scores, but it has laid the foundations for discussions about minimising intervention and thereby admissions. The second RCT, from the UK, compared outpatient care using an ambulatory device to standard care and showed that the median hospital stay was reduced to 0 days compared 4 days in the standard care arm.^15^ The newly updated BTS guidelines, therefore, now prioritise patient symptom burden, rather than pneumothorax size, for PSP encouraging conservative or ambulatory management.^18^

This study has several limitations. First, the data only provide information on patients admitted to the hospital, so they do not cover patients treated for pneumothorax in primary care, ED only, or at home, making it impossible to disentangle changes in incidence from changes in management. Another limitation is the retrospective analysis of the administration data which depend upon accurate and consistent coding practice. The processing cycle of HES and data quality checks are documented elsewhere.^19^ Additionally, the analysis relies on the accuracy of coding for each hospital episode; fortunately, spontaneous pneumothorax is covered by relatively few ICD codes that are specific to this condition. To define SSP, we examined the concurrently-recorded comorbidities and traced back through a ten-year disease history and one subsequent month, which helped us better identify potential pneumothorax cases that might have developed due to other chronic lung diseases. However, other diagnoses listed alongside pneumothorax may be less reliably recorded, making it harder to distinguish between particular spontaneous pneumothorax subtypes.

In conclusion, this study provides contemporary information regarding the recent trends in inpatient-treated spontaneous pneumothorax including the COVID-19 pandemic. There was a significant peak of COVID-19 related pneumothoraces in the later part of 2020 and early 2021. Despite a previous report of increasing incidence of (non-COVID-related) hospitalised spontaneous pneumothorax over the long-term between 1968 and 2016, we did not observe any continued increase throughout this study period. Further work is required to determine whether the change in recent guidelines will further reduce the inpatient management of pneumothorax.

## Contributors

All authors included on the paper fulfil the criteria of authorship. All authors (RH, XZ, RG and EJAM) contributed to study concept, design and review of the manuscript. RG and XZ conducted the statistical methodology. RH conducted the literature search. XZ conducted the data analysis and generated the figures. RG, XZ and RH drafted the manuscript. RG is the guarantor.

## Data sharing statement

RG and XZ had full access to all the data in the study and take responsibility for the integrity of the data and the accuracy of the data analysis.

## Declaration of interests

None to declare.
