# Supplementary material for: Trends in incidence of pneumothorax in England before, during and after the COVID-19 pandemic (2017–2023): a population-based observational study
Source: Lancet Reg Health Eur. 2024 Jul 1;44:100994. doi: 10.1016/j.lanepe.2024.100994 (PMC11267084; doi:10.1016/j.lanepe.2024.100994)
Supplement: Supplementary materials [file mmc1.docx]

**Supplementary materials**

**Table count: 3**

**Figure count: 4**

[**eTable 1 ICD-10 codes for any diagnosis of chronic lung disease** 2](#_Toc162617902)

[**eTable 2 Spontaneous Pneumothorax Inpatient Admissions (Principal diagnosis only) in England from Jan 2017 - Mar 2023, 15 Years and Older, by Age Group and Sex** 3](#_Toc162617903)

[**eTable 3 Underlying IRR for Figure 3&4** 4](#_Toc162617904)

[**eFigure 1. Hospitalisation Rates for Spontaneous Pneumothorax in England (From Jan 2017 to March 2023, spontaneous pneumothorax could be any diagnosis (primary/non-primary))** 15](#_Toc165903428)

[**eFigure 2. Hospitalisation Rates for Spontaneous Pneumothorax in England (From Jan 2017 to March 2023, spontaneous pneumothorax was the principal diagnosis only)** 17](#_Toc165903429)

[**eFigure 3 Incidence Rate Ratio Comparing Spontaneous Pneumothorax Hospitalisation Rates Across Pre-pandemic, Pandemic, and Post-pandemic Periods, spontaneous pneumothorax could be any diagnosis (primary/non-primary))** 19](#_Toc165903430)

[**eFigure 4 Incidence Rate Ratio Comparing Spontaneous Pneumothorax Hospitalisation Rates Across Pre-pandemic, Pandemic, and Post-pandemic Periods, ,spontaneous pneumothorax was the principal diagnosis only** 21](#_Toc165903431)

**eTable 1 ICD-10 codes for any diagnosis of chronic lung disease**

| Tuberculosis | A150, A151, A152, A153, A155, A156, A157, A158, A159, A160, A161, A162, A164, A165, A168, A169, B909 |
| --- | --- |
| Malignancy | C340, C341, C342, C343, C348, C349, C780, D020, D022, D023, D143, D381, D382, Z851, Z852 |
| Sarcoidosis | D860, D861, D862, D863, D868, D869 |
| Cystic Fibrosis | E840, E841, E848, E849 |
| COPD/Emphysema | J40X, J410, J411, J42X, J430, J431, J432, J438, J439, J440, J441, J448, J449 |
| Asthma | J450, J451, J458, J459, J47X |
| Interstitial lung disease | J700, J701, J703, J704, J708, J709, J840, J841, J848, J849, J961, J991, J998 |

**eTable 2 Spontaneous Pneumothorax Inpatient Admissions (Principal diagnosis only) in England from Jan 2017 - Mar 2023, 15 Years and Older, by Age Group and Sex**

|  | **Overall Spontaneous Pneumothorax** | | **Primary Spontaneous Pneumothorax** | | **Secondary Spontaneous Pneumothorax** | | **COVID-related Spontaneous Pneumothorax** | |
| --- | --- | --- | --- | --- | --- | --- | --- | --- |
|  | No. of Admissions | Mean age (SD) | No. of Admissions | Mean age (SD) | No. of Admissions | Mean age (SD) | No. of Admissions | Mean age (SD) |
| **Male, overall** | 29429 | 49.2 (22.8) | 13301 | 37.9 (20.0) | 15733 | 58.5 (20.6) | 395 | 57.9 (20.9) |
| **Male, by Age Group, y** |  | | | | | | | |
|  | No. of Admissions | % | No. of Admissions | % | No. of Admissions | % | No. of Admissions | % |
| 15-34 | 10431 | 35.9 | 7498 | 57.5 | 2858 | 18.3 | 75 | 19.0 |
| 35-49 | 4307 | 14.8 | 2404 | 18.4 | 1857 | 11.9 | 46 | 11.6 |
| 50-64 | 4546 | 15.6 | 1258 | 9.6 | 3201 | 20.5 | 87 | 22.0 |
| ≥65 | 9794 | 33.7 | 1884 | 14.4 | 7723 | 49.4 | 187 | 47.3 |
|  | No. of Admissions | Mean age (SD) | No. of Admissions | Mean age (SD) | No. of Admissions | Mean age (SD) | No. of Admissions | Mean age (SD) |
| **Female, overall** | 11138 | 56.2 (20.8) | 4044 | 46.0 (21.2) | 6966 | 62.1 (18.2) | 128 | 61.0 (19.8) |
| **Female, by Age Group, y** |  | | | | | | | |
|  | No. of Admissions | % | No. of Admissions | % | No. of Admissions | % | No. of Admissions | % |
| 15-34 | 2272 | 20.5 | 1490 | 37.2 | 765 | 11.0 | 17 | 13.3 |
| 35-49 | 1937 | 17.5 | 1056 | 26.3 | 861 | 12.4 | 20 | 15.6 |
| 50-64 | 2073 | 18.7 | 549 | 13.7 | 1501 | 21.6 | 23 | 18.0 |
| ≥65 | 4809 | 43.4 | 914 | 22.8 | 3827 | 55.0 | 68 | 53.1 |

**eTable 3 Underlying IRR for Figure 3&4**

Figure 3b.

| Time | IRR | IRR Upper | IRR Lower | Group |
| --- | --- | --- | --- | --- |
| pre | 1 | 1 | 1 | Overall |
| Mar_2020 | 0.928 | 0.811 | 1.057 | Overall |
| Apr_2020 | 1.059 | 0.933 | 1.198 | Overall |
| May_2020 | 0.918 | 0.801 | 1.048 | Overall |
| Jun_2020 | 0.982 | 0.859 | 1.116 | Overall |
| Jul_2020 | 0.964 | 0.842 | 1.097 | Overall |
| Aug_2020 | 0.987 | 0.863 | 1.123 | Overall |
| Sep_2020 | 0.939 | 0.818 | 1.072 | Overall |
| Oct_2020 | 1.134 | 0.999 | 1.282 | Overall |
| Nov_2020 | 1.102 | 0.969 | 1.249 | Overall |
| Dec_2020 | 1.22 | 1.077 | 1.376 | Overall |
| Jan_2021 | 1.652 | 1.48 | 1.84 | Overall |
| Feb_2021 | 1.145 | 1.007 | 1.297 | Overall |
| post | 1.063 | 0.985 | 1.146 | Overall |
| pre | 1 | 1 | 1 | PSP |
| Mar_2020 | 0.74 | 0.611 | 0.886 | PSP |
| Apr_2020 | 0.75 | 0.62 | 0.899 | PSP |
| May_2020 | 0.872 | 0.73 | 1.033 | PSP |
| Jun_2020 | 0.915 | 0.768 | 1.08 | PSP |
| Jul_2020 | 1.006 | 0.851 | 1.182 | PSP |
| Aug_2020 | 0.859 | 0.717 | 1.021 | PSP |
| Sep_2020 | 0.95 | 0.799 | 1.122 | PSP |
| Oct_2020 | 0.912 | 0.764 | 1.081 | PSP |
| Nov_2020 | 0.805 | 0.667 | 0.964 | PSP |
| Dec_2020 | 0.876 | 0.73 | 1.043 | PSP |
| Jan_2021 | 0.75 | 0.616 | 0.905 | PSP |
| Feb_2021 | 0.832 | 0.69 | 0.996 | PSP |
| post | 0.932 | 0.846 | 1.027 | PSP |
| pre | 1 | 1 | 1 | SSP |
| Mar_2020 | 0.748 | 0.66 | 0.845 | SSP |
| Apr_2020 | 0.727 | 0.639 | 0.822 | SSP |
| May_2020 | 0.815 | 0.722 | 0.917 | SSP |
| Jun_2020 | 0.961 | 0.858 | 1.071 | SSP |
| Jul_2020 | 0.894 | 0.795 | 1.001 | SSP |
| Aug_2020 | 1.039 | 0.932 | 1.156 | SSP |
| Sep_2020 | 0.834 | 0.739 | 0.938 | SSP |
| Oct_2020 | 0.964 | 0.86 | 1.076 | SSP |
| Nov_2020 | 0.817 | 0.723 | 0.921 | SSP |
| Dec_2020 | 0.841 | 0.745 | 0.947 | SSP |
| Jan_2021 | 0.766 | 0.675 | 0.867 | SSP |
| Feb_2021 | 0.832 | 0.735 | 0.937 | SSP |
| post | 0.952 | 0.892 | 1.016 | SSP |

Figure 4b

| Time | IRR | IRR Upper | IRR Lower | Group |
| --- | --- | --- | --- | --- |
| pre | 1 | 1 | 1 | Overall |
| Mar_2020 | 0.764 | 0.657 | 0.884 | Overall |
| Apr_2020 | 0.708 | 0.604 | 0.823 | Overall |
| May_2020 | 0.794 | 0.684 | 0.917 | Overall |
| Jun_2020 | 0.855 | 0.74 | 0.983 | Overall |
| Jul_2020 | 0.867 | 0.75 | 0.996 | Overall |
| Aug_2020 | 0.896 | 0.776 | 1.028 | Overall |
| Sep_2020 | 0.879 | 0.76 | 1.01 | Overall |
| Oct_2020 | 0.93 | 0.807 | 1.067 | Overall |
| Nov_2020 | 0.81 | 0.696 | 0.937 | Overall |
| Dec_2020 | 0.826 | 0.71 | 0.955 | Overall |
| Jan_2021 | 0.838 | 0.721 | 0.969 | Overall |
| Feb_2021 | 0.829 | 0.713 | 0.96 | Overall |
| post | 0.962 | 0.89 | 1.04 | Overall |
| pre | 1 | 1 | 1 | PSP |
| Mar_2020 | 0.74 | 0.593 | 0.911 | PSP |
| Apr_2020 | 0.582 | 0.454 | 0.735 | PSP |
| May_2020 | 0.775 | 0.623 | 0.952 | PSP |
| Jun_2020 | 0.804 | 0.648 | 0.985 | PSP |
| Jul_2020 | 0.887 | 0.721 | 1.078 | PSP |
| Aug_2020 | 0.711 | 0.565 | 0.883 | PSP |
| Sep_2020 | 0.954 | 0.78 | 1.155 | PSP |
| Oct_2020 | 0.822 | 0.662 | 1.009 | PSP |
| Nov_2020 | 0.743 | 0.591 | 0.921 | PSP |
| Dec_2020 | 0.804 | 0.645 | 0.991 | PSP |
| Jan_2021 | 0.718 | 0.568 | 0.895 | PSP |
| Feb_2021 | 0.769 | 0.612 | 0.953 | PSP |
| post | 0.921 | 0.824 | 1.03 | PSP |
| pre | 1 | 1 | 1 | SSP |
| Mar_2020 | 0.768 | 0.658 | 0.89 | SSP |
| Apr_2020 | 0.736 | 0.628 | 0.855 | SSP |
| May_2020 | 0.796 | 0.683 | 0.921 | SSP |
| Jun_2020 | 0.882 | 0.763 | 1.015 | SSP |
| Jul_2020 | 0.86 | 0.742 | 0.991 | SSP |
| Aug_2020 | 1.024 | 0.893 | 1.169 | SSP |
| Sep_2020 | 0.835 | 0.718 | 0.965 | SSP |
| Oct_2020 | 0.969 | 0.841 | 1.111 | SSP |
| Nov_2020 | 0.817 | 0.7 | 0.947 | SSP |
| Dec_2020 | 0.82 | 0.703 | 0.951 | SSP |
| Jan_2021 | 0.773 | 0.66 | 0.901 | SSP |
| Feb_2021 | 0.804 | 0.688 | 0.935 | SSP |
| post | 0.953 | 0.88 | 1.033 | SSP |


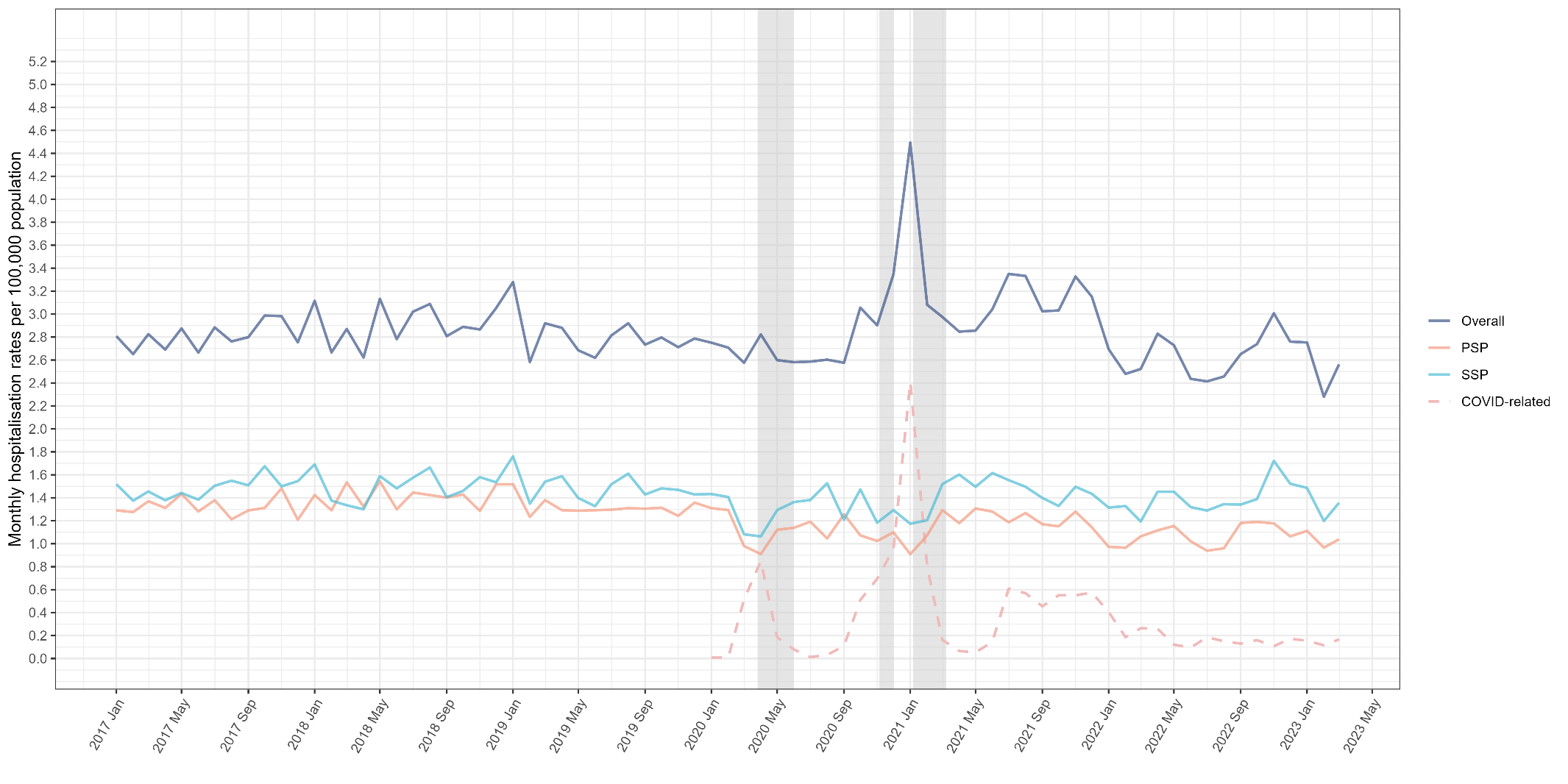


1. Male


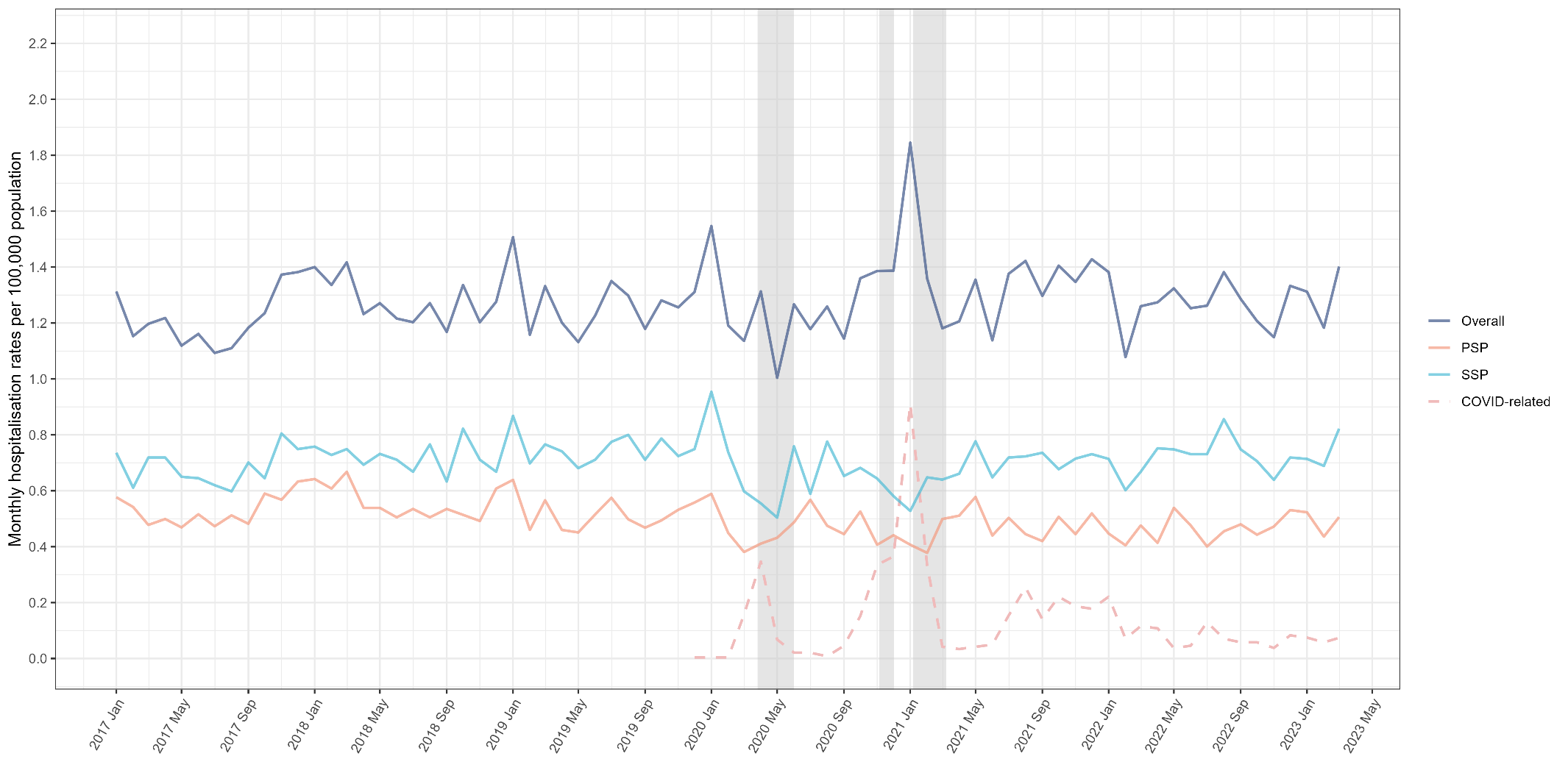


1. Female

**eFigure 1. Hospitalisation Rates for Spontaneous Pneumothorax in England (From Jan 2017 to March 2023, spontaneous pneumothorax could be any diagnosis (primary/non-primary))**

1. *Hospitalisation Rates for Spontaneous Pneumothorax in Male. b. Hospitalisation Rates for Spontaneous Pneumothorax in Female.*


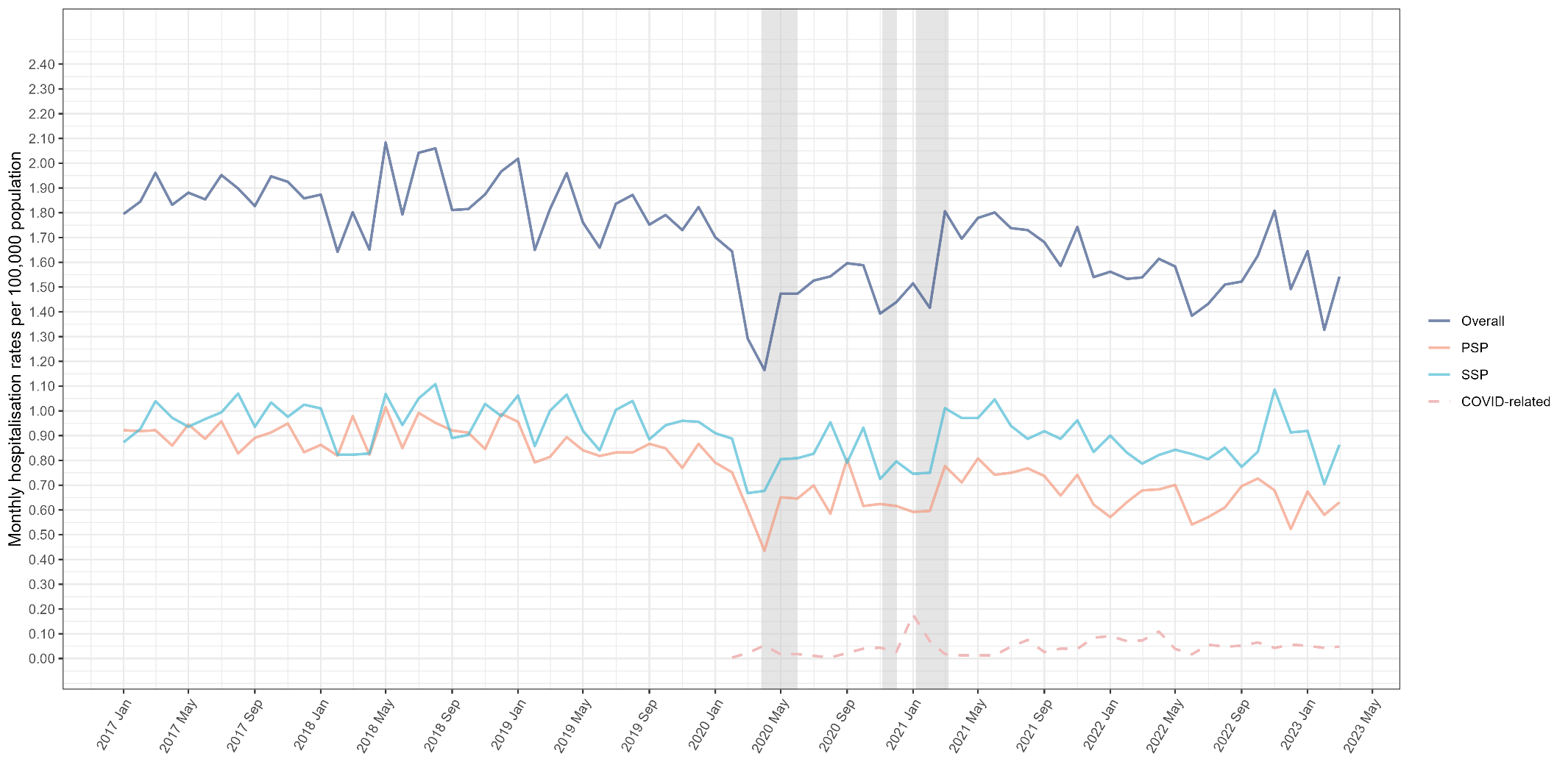


1. Male


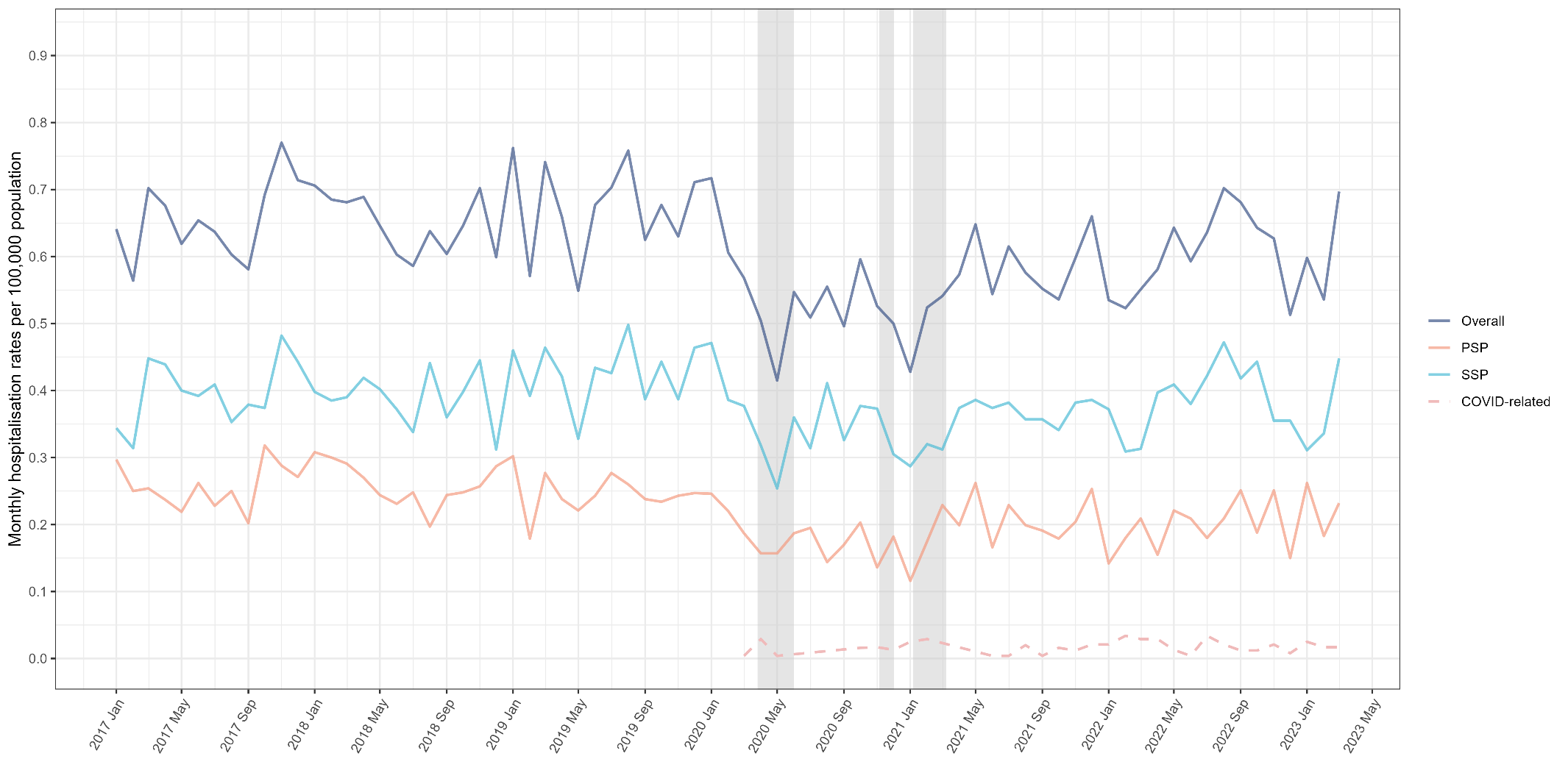


1. Female

**eFigure 2. Hospitalisation Rates for Spontaneous Pneumothorax in England (From Jan 2017 to March 2023, spontaneous pneumothorax was the principal diagnosis only)**

1. *Hospitalisation Rates for Spontaneous Pneumothorax in Male. b. Hospitalisation Rates for Spontaneous Pneumothorax in Female.*


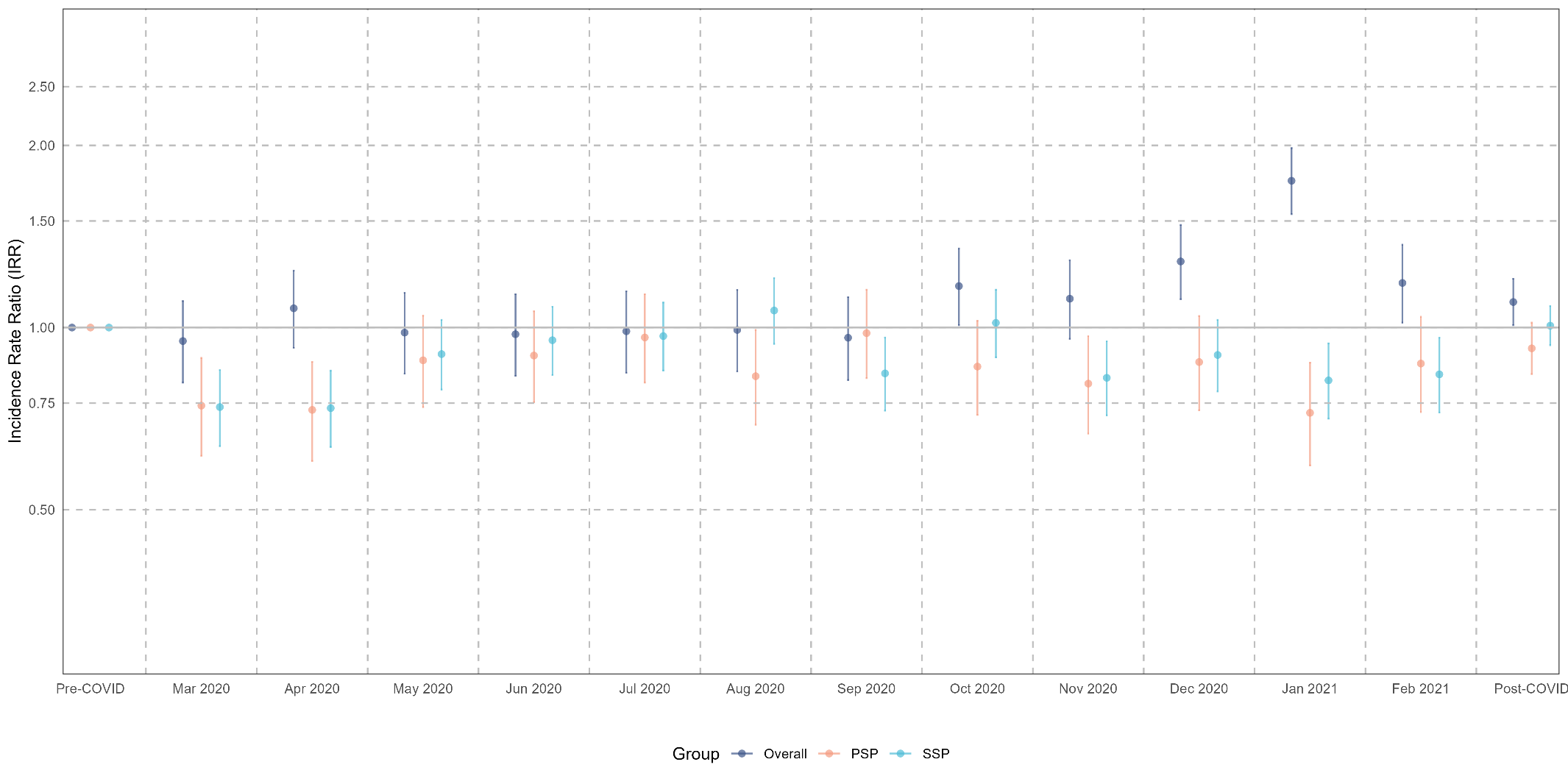


1. Male


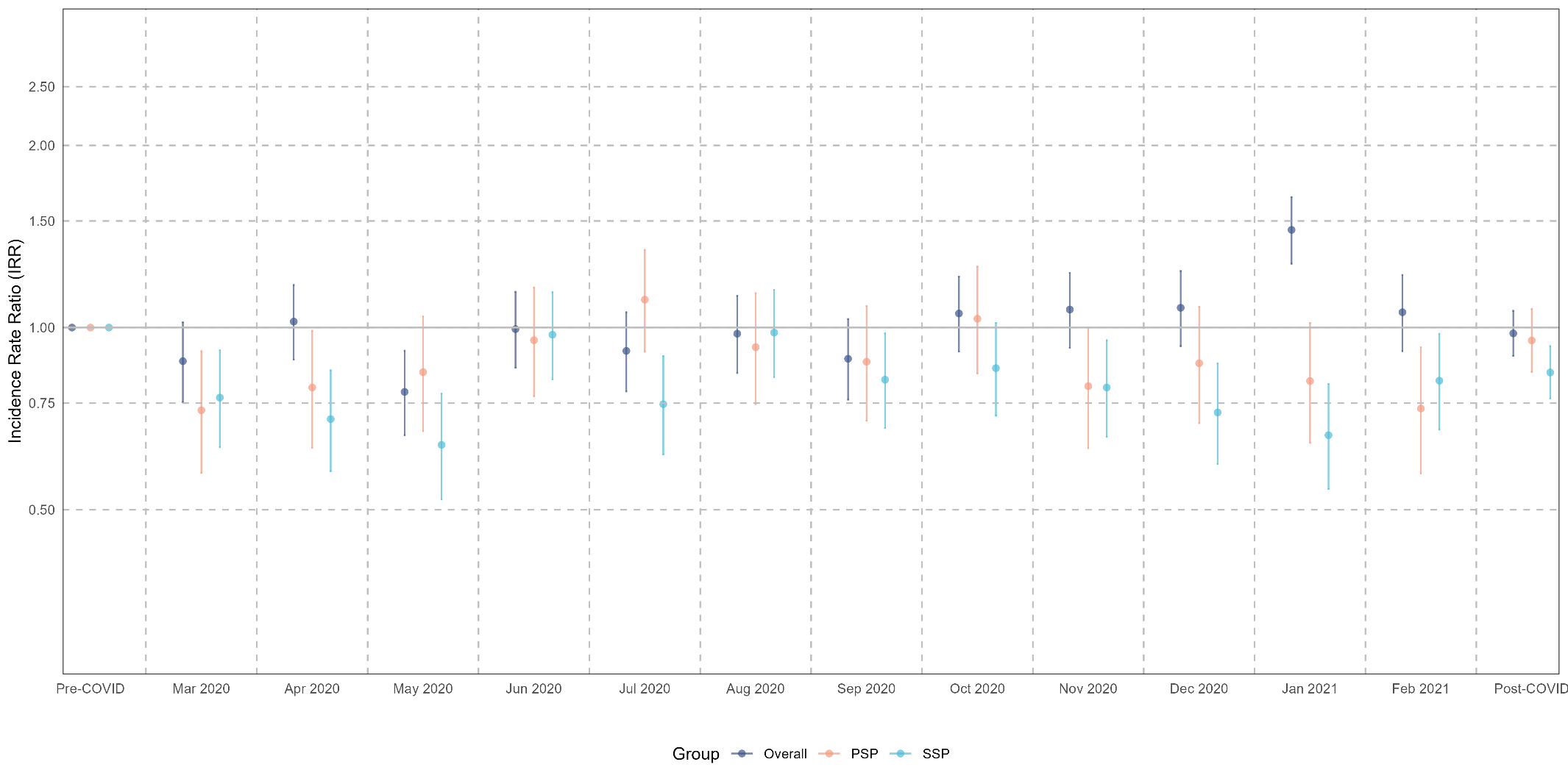


1. Female

**eFigure 3 Incidence Rate Ratio Comparing Spontaneous Pneumothorax Hospitalisation Rates Across Pre-pandemic, Pandemic, and Post-pandemic Periods, spontaneous pneumothorax could be any diagnosis (primary/non-primary))**


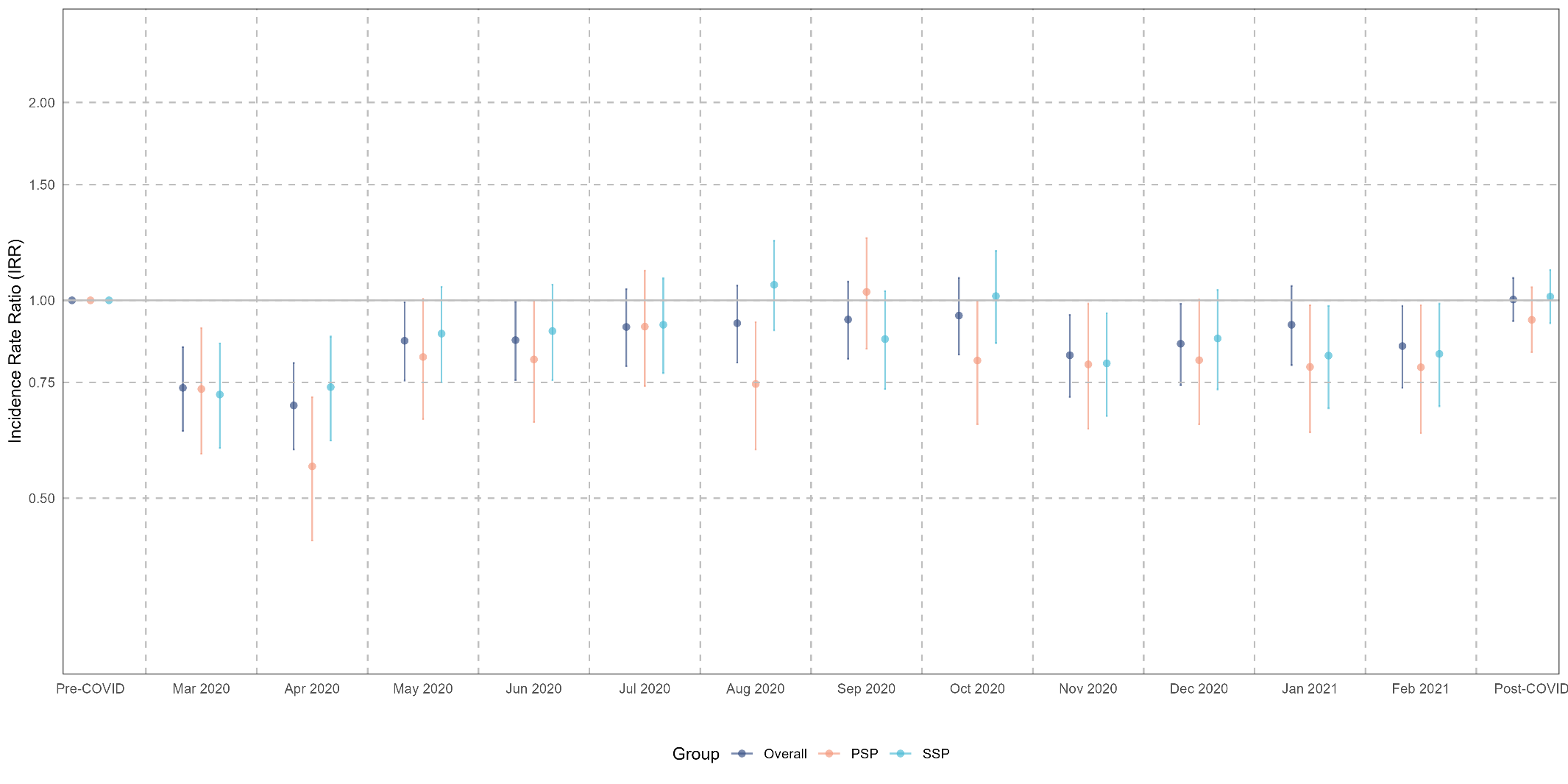


1. Male


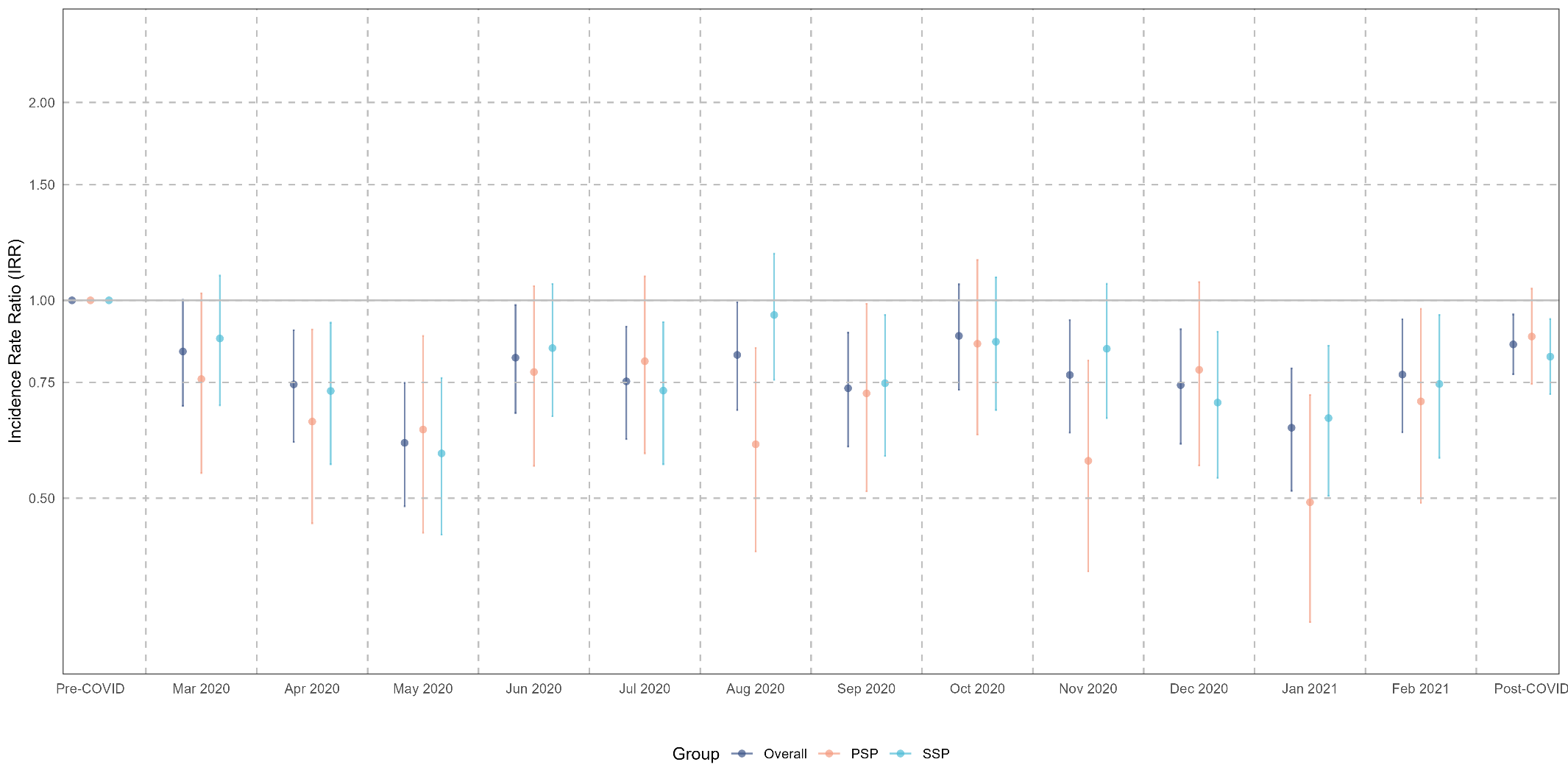


1. Female

**eFigure 4 Incidence Rate Ratio Comparing Spontaneous Pneumothorax Hospitalisation Rates Across Pre-pandemic, Pandemic, and Post-pandemic Periods, ,spontaneous pneumothorax was the principal diagnosis only**
